# PHI: a Galaxy-based workflow for reproducible prophage-host interaction analysis and standardized viral-genomics reporting

**DOI:** 10.1186/s12859-026-06711-w

**Published:** 2026-11-11

**Authors:** João Pedro Saraiva, Felipe Borim Corrêa, Matthias Bernt, Nawras Ghanem, Esteban Nieto, Rodolfo Brizola Toscan, Lukas Y. Wick, Antonis Chatzinotas

**Affiliations:** 1 Department of Applied Microbial Ecology, Helmholtz Centre for Environmental Research - UFZ, Leipzig, 04318 Germany; 2 Department of Computational Biology and Chemistry, Helmholtz Centre for Environmental Research - UFZ, Leipzig, 04318 Germany; 3 Department of Biochemistry and Biotechnology, University of Thessaly, Viopolis, Larissa, 41500 Greece; 4 Małopolska Centre of Biotechnology, Jagiellonian University, Kraków, Poland; 5 Institute of Biology, Leipzig University, Leipzig, 04103 Germany; 6 German Centre for Integrative Biodiversity Research (iDiv) Halle-Jena- Leipzig, Leipzig, 04103 Germany

**Keywords:** Bioinformatics, Galaxy-server, Virology, Host-phage interactions

## Abstract

**Background:**

Viruses that infect bacteria, known as bacteriophages or phages, are widespread in nature and play important roles in shaping microbial communities and ecosystem functions. Some phages can integrate into bacterial genomes as “prophages”, where they may influence the biology of their host by carrying genes that affect metabolism, virulence, or environmental adaptation. Despite their importance, studying prophages and their interactions with bacterial hosts remains challenging because it typically requires combining many complex computational tools and can be resource-intensive.

**Results:**

In this study, we introduce the Prophage-Host Interaction Toolkit (PHI), a user-friendly and automated workflow available through the Galaxy platform. PHI brings together multiple established tools into a single, reproducible pipeline that identifies candidate prophages, evaluates their quality, predicts host relationships, and characterizes key functional genes. Importantly, all results are summarized in an interactive report that simplifies interpretation. Applied to the MBARC22 mock community of 22 bacterial reference genomes as a workflow demonstration, PHI identified 41 candidate prophages across 14 hosts, including high- and medium-quality viral genomes. Most host genomes exhibited > 99% completeness and < 1% contamination, while DefenseFinder identified between 3 and 24 antiviral defense systems per genome. To further evaluate applicability to metagenome-derived assemblies, PHI was also applied to three MAGs and their matched reference genomes. Differences in candidate prophage counts and sequence-level correspondence between MAGs and references demonstrated that prophage recovery can be strongly influenced by genome fragmentation and assembly continuity, even in otherwise high-quality MAGs.

**Conclusions:**

By removing installation barriers and consolidating the outputs of multiple established tools, PHI lowers the barrier to advanced phage analysis, enabling both specialists and non-experts to explore phage-host interactions and their implications in areas such as microbiome research, biotechnology, and environmental science.

**Supplementary Information:**

The online version contains supplementary material available at https://doi.org/10.1186/s12859-026-06711-w.

## Background

Phages are viruses that infect bacterial cells. After infection, lytic viruses can replicate while lysogenic phages can integrate their genome into the chromosome of their bacterial hosts [1]. Prophages often carry auxiliary metabolic genes (AMGs) that affect host metabolic and broader ecosystem functions [2, 3]. The automated discovery and comprehensive profiling (e.g., taxonomic assignment, functional annotation, and life-cycle prediction) of phages within host genomes remains challenging due to the complexity of building bioinformatic pipelines and useful summaries and visualizations of their results. Existing workflows typically focus on either identification and gene annotation [4, 5] or specific interaction aspects [6], lacking a comprehensive approach that addresses a wide spectrum of phage-host relationships within genomic data.

Several methods and workflows have advanced the field of phage identification, host prediction and viral genome annotation, such as PHASTEST [5], VirSorter2 [7], CHERRY [8], VIRify [4], and CPT Phage workflows (https://usegalaxy.eu/published/workflow?id=0b9071d5dfa38559 and https://usegalaxy.eu/published/workflow?id=a644e6b450dabca8) hosted in the Galaxy server. However, they often focus on specific tasks providing limited information on both host and phage perspectives, require extensive computational resources, or lack user-friendly interfaces, thereby limiting their use by the scientific community.

We present the Prophage-Host Interaction Toolkit (PHI), a comprehensive and automated framework within the Galaxy [9] platform (e.g., publicly usable via https://usegalaxy.eu), for the identification and profiling of phages within host genomes that unifies established tools into a reproducible workflow. PHI overcomes the limitations of existing tools, such as the complexity and difficulty that arise with installing and running multiple tools. PHI also compiles all results in a single report, facilitating knowledge extraction and enabling researchers from diverse backgrounds to benefit from bioinformatics applications without requiring extensive technical training.

Key features of PHI include its modular architecture, allowing for easy updates and customization. PHI incorporates tools for reproducible detection and contextual profiling of candidate prophages, identification of phage-host pairs, and integration of iPHoP-derived host-prediction evidence (which combines multiple host-association signals, including CRISPR spacer matches, sequence similarity, and sequence-composition-based evidence). Furthermore, it provides comprehensive visualization tools for depicting phage genomic features and host interaction networks, facilitating the interpretation of complex data.

The following sections describe the PHI toolkit, including the integrated tools and its application to a mock-community case study, demonstrating its utility for reproducible workflow execution and integrated reporting.

## Implementation

### Workflow

The workflow is implemented in Galaxy [9] and it is publicly available through the Galaxy Europe server (https://usegalaxy.eu/published/workflow?id=c62d65832377e376). To achieve the full functionality described in this manuscript, we pre-tailored the pipeline with established tools and analyses that generate an HTML report through an R Markdown script. Additionally, the flexibility of Galaxy allows for full customization of the pipeline. Any modification or expansion of the pipeline is possible as long as the desired tools are available on the server and the code used to generate the report is updated.

### Pipeline description

**Fig. 1:**
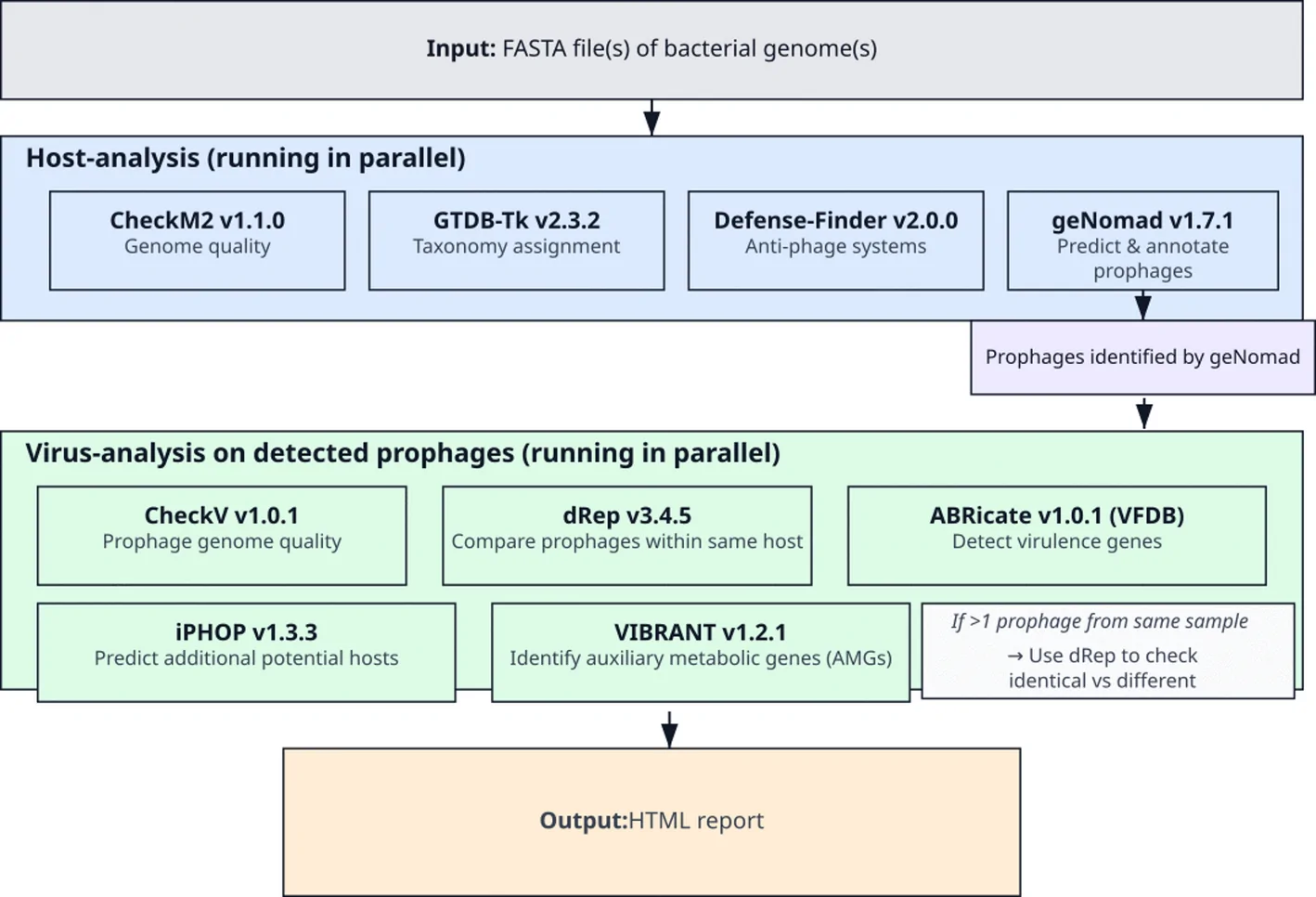
Workflow of PHI toolkit. The user submits one or more FASTA files for processing. The pipeline begins with the parallel execution of the host analysis tools for each submitted genome. Prophages identified by geNomad [14] are then submitted to several virus analysis tools (which are also executed in parallel). If no candidate prophage or viral sequence is identified for a genome, downstream viral analyses are skipped for that genome and the final report records the absence of viral outputs. To generate the HTML report, a script written in R Markdown renders an interactive and user-friendly HTML document. This script first loads several key outputs for each tool, plus the input host genomes. Host genome analysis includes: the geNomad summary table (*_virus_summary.tsv), which reports detected prophages within bacterial genomes; the CheckM2 quality report (quality_report.tsv), which contains completeness and contamination estimates; the taxonomic classification from GTDB-Tk (gtdbtk.bac120.summary.tsv); and the predicted defense systems identified by DefenseFinder (*_defense_finder_systems.tsv). Prophage genome analysis includes: the VIBRANT annotations of auxiliary metabolic genes (VIBRANT_AMG_individuals_*_virus.tsv); the CheckV quality summary file (quality_summary.tsv); the host prediction results from iPHoP (Host_prediction_to_genome_m90.csv); the prophage clustering and comparison output from dRep (Cdb.csv) and the clustering dendrogram (Primary_clustering_dendrogram.pdf); and the virulence factor annotations generated by Abricate (*_virus_vfdb.tsv)

A simplified schematic of the workflow is shown in Fig. 1. A more detailed workflow schema produced by Galaxy’s workflow editor is available in the Additional File 1: Figure S1. The PHI pipeline accepts bacterial genomes in FASTA format as input files. These genomes are submitted in parallel to the following host-analysis tools: CheckM2 1.0.2 + galaxy1 using the CheckM diamond DB [10] to assess the quality, GTDB-Tk 2.4.1 + galaxy0 using the full DB release 220 [11, 12] to assign taxonomy, Defense-Finder 2.0.1 + galaxy1 using models 2.0.2 [13] to detect known anti-phage systems, and finally geNomad 1.11.1 + galaxy0 using reference data version 1.9 [14] to predict and annotate prophages. Once a genome has been processed and prophages have been identified by geNomad, several virus-analysis tools also run in parallel, which are: (i) CheckV 1.0.3 + galaxy0 using reference data version 1.5 [15] to evaluate the quality of prophage genomes; (ii) dRep compare 3.6.2 + galaxy1 using an ANI threshold of 0.95 [16] to cluster and compare prophage genomes recovered from the same host, thereby distinguishing identical from distinct prophage sequences; (iii) ABRicate 1.0.1 [17] using the Virulence Factor Database (VFDB) [18] to identify virulence genes in the predicted prophage genomes; (iv) iPHoP 1.3.3 + galaxy0 [6] to predict other potential hosts; and, (v) VIBRANT 1.2.1 + galaxy2 using reference data version 1.2.1 [19] to identify auxiliary metabolic genes (AMGs) in the prophages. Unless stated otherwise, default parameters were used. The dRep ANI threshold to form secondary clusters was set to 0.95 instead of the default 0.99 to decrease clustering stringency. The database used by ABRicate was set to VFDB instead of the default ResFinder since we are interested in virulence factors. Within the Galaxy environment, several tools are available for viral and prophage identification, including VirSorter2 and VIBRANT. VirSorter2 uses multiple classifiers based on viral genomic features and profile-HMM information and is designed for broad detection of diverse viral groups, whereas VIBRANT combines protein-based signatures and machine-learning approaches with downstream viral functional characterization. CheckV and VIBRANT can also identify or delineate prophage regions, but we decided to select geNomad as the primary prophage detection method because it combines sequence- and gene-content-based classification with a dedicated approach for detecting integrated proviral regions, performs well on both complete and fragmented sequences, is actively maintained, is available in the Galaxy Europe environment, and produces structured outputs that can be consistently integrated with downstream PHI modules. Restricting primary prophage detection to a single standardized module also avoids combining partially overlapping predictions generated using different algorithms and thresholds. The workflow concludes with the automated report generation via R Markdown, producing a structured HTML summary (Additional File 2: Figure S2). The generated HTML report is organized into multiple thematic sections that integrate host, prophage, and interaction-level analyses. In addition to summary tables of genome quality, taxonomy, and prophage content, the report includes interactive visualizations such as host-virus association networks, iPHoP-derived host-prediction outputs, and genome-level feature maps. These components allow users to explore relationships between prophages and their predicted hosts, assess host range, and evaluate genomic context in a unified framework. Tool versions, parameters, and database releases are documented inside the workflow run to ensure reproducibility. A step-by-step usage instructions manual is available in Additional File 3: Text S1).

### Workflow decision logic and edge-case handling

PHI first runs host-level analyses on all submitted genome FASTA files and then triggers downstream viral analyses only when geNomad reports candidate viral or prophage sequences. If no candidate viral sequences are recovered for a genome, the downstream viral modules (CheckV, dRep, ABRicate/VFDB, iPHoP, and VIBRANT) are skipped for that genome and the final report indicates that no viral output was available. If only one or too few viral sequences are available, dereplication or clustering outputs are not interpreted as comparative evidence. Missing module outputs are reported as unavailable rather than silently omitted. These rules make the workflow transparent and reproducible, but they do not modify the underlying predictions generated by each integrated tool.

To calculate the quality classes for host genomes that are displayed on the Overview Table, we used the quality definitions described by the Minimum Information about a Single Amplified Genome (MISAG)/ Minimum Information about a Metagenome-Assembled Genome (MIMAG) standards [20]. ‘High-quality draft’ indicates that a Single Amplified Genome (SAG) or Metagenome-Assembled Genome (MAG) is > 90% complete with less than 5% contamination. ‘Medium-quality draft’ SAGs and MAGs are those genomes with completeness estimates of ≥ 50% and less than 10% contamination. All other SAGs and MAGs (< 50% complete with < 10% contamination) should be reported as ‘low-quality drafts’ [21]. The quality score is defined as completeness – 5* contamination [22].

### Demonstration dataset

To demonstrate workflow execution, output integration, and report interpretability, we applied PHI to 22 bacterial genomes from a defined mock community dataset [23]. This community is routinely used as a baseline for testing bioinformatics analyses and data interpretation. As we were focusing on phages, we downloaded the FASTA sequences of the complete genomes of 22 bacterial strains from the National Centre for Biotechnology Information (NCBI), except for the reference genome NC_010067.1, which was marked as “Record suppressed”. The mock community is hereafter named as MBARC22. The accession numbers for each genome are shown in the HTML report (Additional File 2: Figure S2).

## Results

### Workflow-level runtime and resource usage

The total runtime for the MBARC22 dataset was approximately 3.6 h. GTDB-Tk required the highest amount of CPU cores (32). In terms of memory, iPHoP was the most demanding, requiring over 130 GB of RAM. In contrast to CheckM and GTDB-Tk which processes all genomes in a single run, all other tools process each genome separately. Of the latter tools, geNomad required the longest time to process a genome on average (21 min) while Abricate was the quickest (nine seconds). The average memory peaks and runtimes are shown in Table 1.

**Table 1:** Average computational resources used by each tool for processing MBARC22

Tool	Average Runtime (s)	Standard deviation (+/-)	Average memory peak (Gb)	Standard deviation (+/-)
**CheckM**	1560	0	11.0	0
**GTDB-Tk**	8700	0	91.8	0
**dRep**	26	11.3	0.407	0.0351
**DefenseFinder**	136.2	64.2	0.957	0.0919
**geNomad**	1260	618	18.2	0.55
**Abricate**	9	6.5	0.270	0.023
**CheckV**	100	56.9	3.5	0.71
**iPHoP**	846	178.8	112.9	21
**VIBRANT**	85.6	108.6	1.80	0.68

The complete values of memory peaks and runtime per run are available in Additional File 4: Table S1. Note that the resource requirements exceed the resources typically available to researchers. Additionally, this resource-use analysis should be interpreted as computational profiling of the workflow rather than as a predictive-performance benchmark of the underlying tools.

To further evaluate scalability, we performed an additional analysis in which individual tool runtimes were grouped by input file-size intervals, and mean runtime values were calculated for each interval (Additional File 5: Table S2). This analysis showed that runtime did not increase linearly with file size for most tools. Lightweight tools such as ABRicate remained below one minute on average across the tested intervals, whereas tools involving larger database searches or more complex sequence characterization, including geNomad, CheckV, and DefenseFinder, showed greater runtime variability. This suggests that runtime is influenced not only by input file size, but also by sequence composition, contig structure, number of detected viral regions, and fixed initialization costs. iPHoP showed relatively stable runtime across the tested small-file intervals but remained the most memory-intensive step, indicating that memory availability rather than runtime is the main practical constraint for full PHI analyses. Batch-level tools such as GTDB-Tk and CheckM2 should be interpreted separately because they process the complete submitted genome set simultaneously rather than individual genome files.

Implementation in Galaxy improves accessibility because usegalaxy.eu provides public access to the required computational infrastructure.

### Host genome analyses

#### Completeness and contamination

**Fig. 2:**
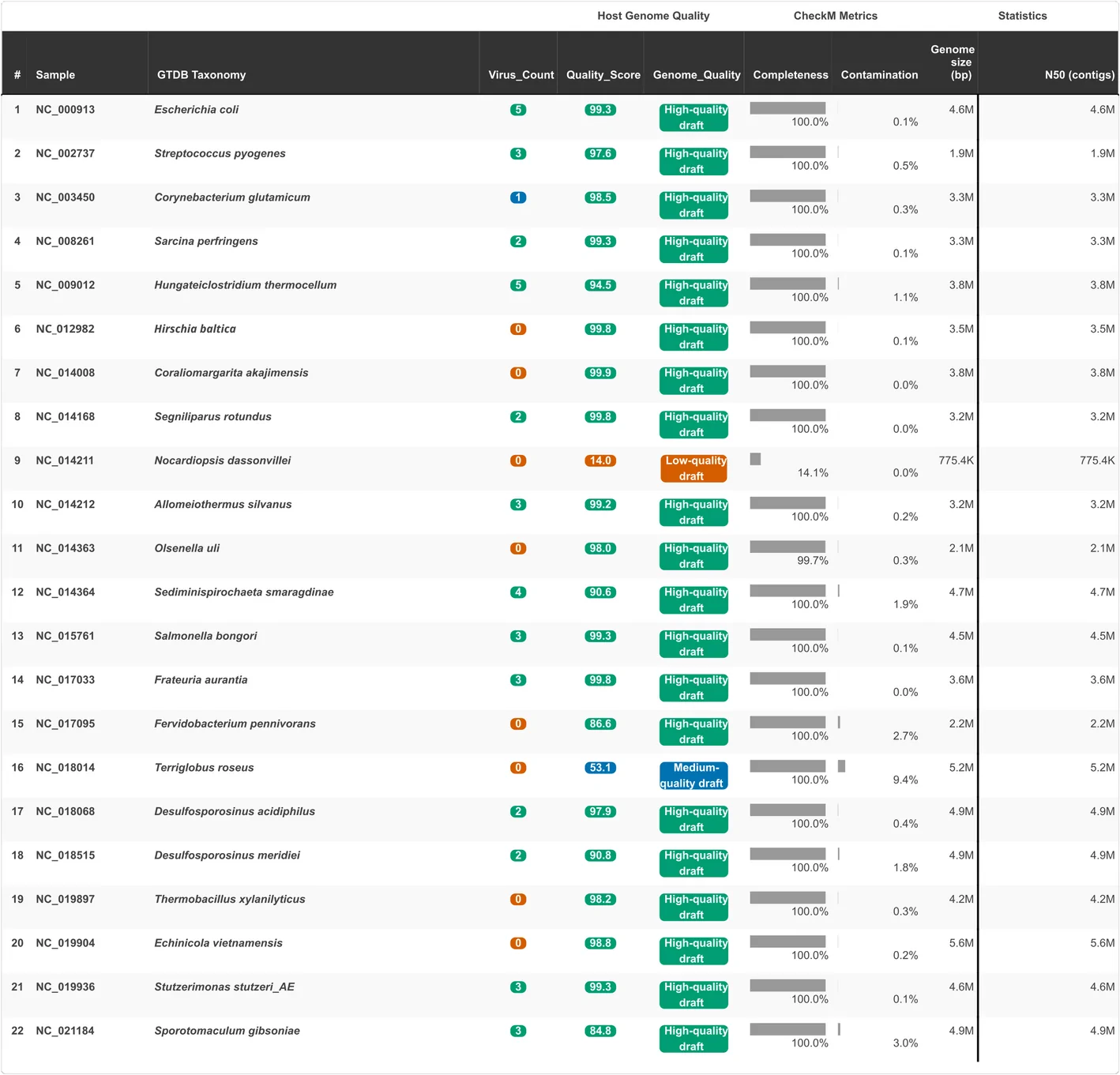
Visualization of the HTML report showing a summary of host genome metrics. The table shows the sample IDs, the taxonomy assignment, the number of viruses identified in the sample, quality metrics such as completeness and contamination, and genome statistics

Except for NC_014008 (14.1% completeness), most bacterial genomes were classified as high-quality drafts (> 90% completeness, < 5% contamination) (Fig. 2).

#### Detection and distribution of antiviral defense systems across samples

PHI integrated DefenseFinder outputs to summarize the presence of prokaryotic antiviral defense systems across multiple genome assemblies (Table 2). The results revealed a diverse array of defense mechanisms distributed unevenly among the samples. The number of detected defense systems per genome ranged from 3 to 24, with NC_021184.1 exhibiting the highest count (24 systems) and NC_014168.1 presenting the fewest (3 systems). In terms of diversity, NC_021184.1 displayed the greatest richness in both system types (13 unique types) and subtypes (*n* = 18), followed by NC_019904.1, which contained 12 system types and 13 subtypes. Conversely, samples such as NC_014168.1 showed limited diversity, with only 2 unique types. Gene content associated with defense systems also varied substantially. The sample NC_021184.1 encoded the largest number of defense-related genes (*n* = 75), while NC_014168.1 had the fewest (*n* = 4). These results illustrate how PHI integrates and reports DefenseFinder outputs to enable comparison of antiviral defense repertoires across genomes. The observed variation highlights the level of detail that can be captured using the DefenseFinder module within the Phage Toolkit. To note, the DefenseFinder output provides host-context information that can help interpret candidate prophage content in relation to bacterial anti-phage defense potential. In the MBARC22 demonstration dataset, variation in the number and diversity of predicted defense systems among host genomes should be interpreted cautiously, because the dataset was not designed to test ecological associations between prophage load and defense repertoires. DefenseFinder predictions indicate the presence of putative defense-system components and do not by themselves demonstrate that a host is resistant to infection, that a prophage is active or inactive, or that a specific defense system interacts with a specific prophage. Such interpretations would require larger comparative datasets, appropriate ecological metadata, and functional validation.

**Table 2:** Example filtered DefenseFinder results presented in the HTML report

System ID	Type	Subtype	Proteins in System	Gene count	Profile names in system
NC_000913.3_Lit_3	Lit	Lit	NC_000913.3_1111	1	Lit__Lit
NC_000913.3_RM_Type_IV_9	RM	RM_Type_IV	NC_000913.3_1130	1	RM_Type_IV__Type_IV_05
NC_000913.3_Hachiman_1	Hachiman	Hachiman	NC_000913.3_2589,NC_000913.3_2590	2	Hachiman__HamA_2,Hachiman__HamB
NC_000913.3_RnlAB_6	RnlAB	RnlAB	NC_000913.3_2591,NC_000913.3_2592	2	RnlAB__RnlA, RnlAB__RnlB
NC_000913.3_CAS_Class1-Subtype-I-E_11	Cas	CAS_Class1-Subtype-I-E	NC_000913.3_2706,NC_000913.3_2707,NC_000913.3_2708,NC_000913.3_2709,NC_000913.3_2710,NC_000913.3_2711,NC_000913.3_2712,NC_000913.3_2713	8	cas1_I-E_1,cas2_I-E_2,cas3_I_5,cas5_I-E_3,cas6e_I_II_III_IV_V_VI_1,cas7_I-E_2,cas8e_I-E_1,cse2gr11_I-E_1
NC_000913.3_MazEF_4	MazEF	MazEF	NC_000913.3_2734,NC_000913.3_2735	2	MazEF__MazE, MazEF__MazF
NC_000913.3_MazEF_5	MazEF	MazEF	NC_000913.3_4140,NC_000913.3_4141	2	MazEF__MazE, MazEF__MazF
NC_000913.3_RM_Type_IV_10	RM	RM_Type_IV	NC_000913.3_4262,NC_000913.3_4263	2	RM_Type_IV__FAM_1,RM_Type_IV__FAM_2
NC_000913.3_RM_Type_I_8	RM	RM_Type_I	NC_000913.3_4265,NC_000913.3_4266,NC_000913.3_4267	3	RM__Type_I_MTases_FAM_2,RM__Type_I_REases_FAM_2.einsi_trimmed, RM__Type_I_S_52

The filtered contents include the identification of defense systems, the type, subtype, and their locations in the contig list

#### Identification and quality of prophages

**Fig. 3:**
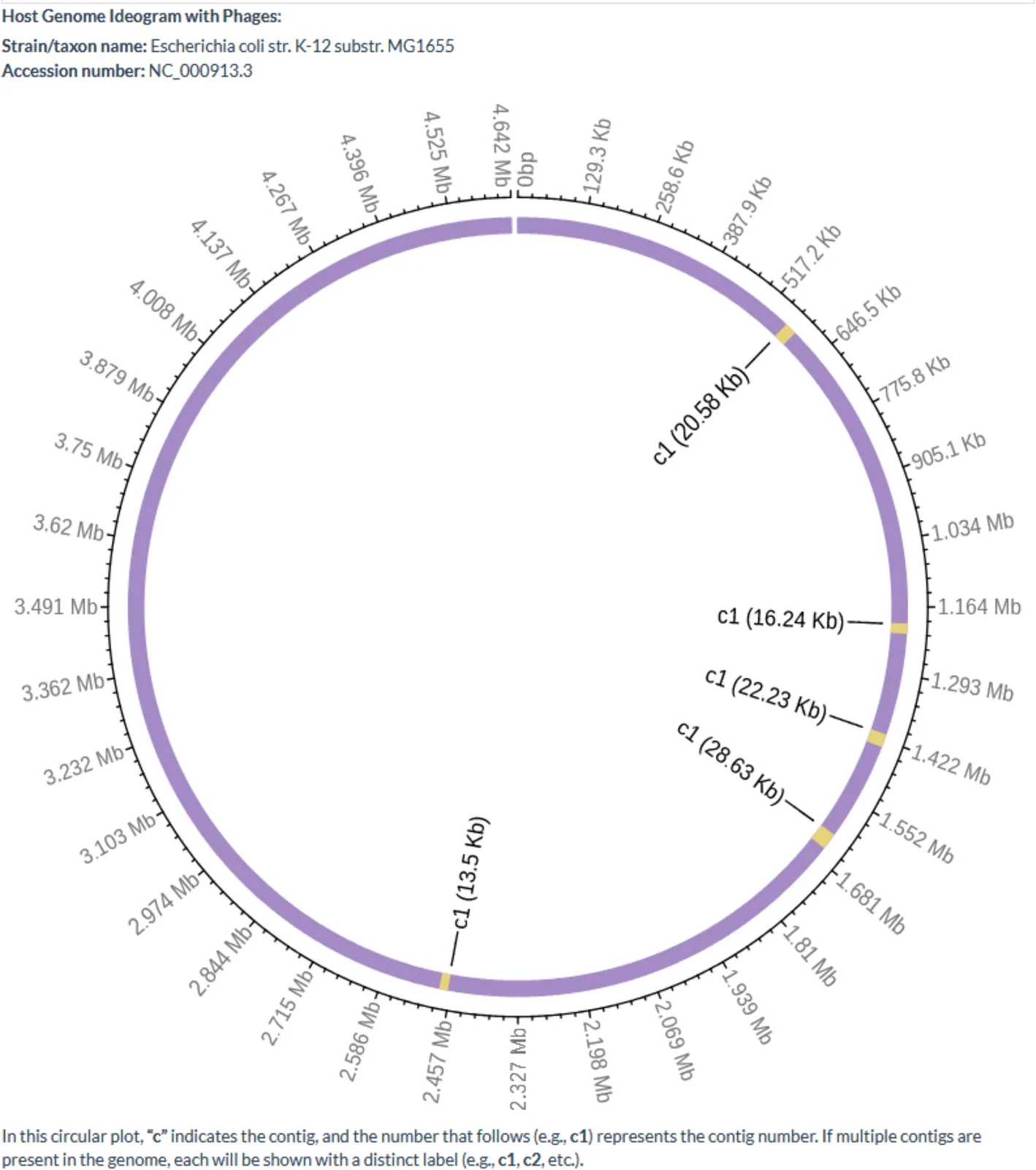
Example host-genome ideogram generated by the PHI HTML report. The ideogram shows the host strain name and corresponding NCBI accession numbers (if available), genomic location and length of candidate prophages predicted in selected MBARC22 host genomes

In the demonstration dataset, the report showed that 14 out of the 22 analyzed bacterial genomes were hosting candidate prophages (Fig. 2). A total of 41 prophages were found in the sample pool, with seven and nine being classified as high- and medium-quality, respectively. The sequences were also categorized using MIUViG standards. Prophages carried varying numbers of genes, typically ranging between 19 and 58 genes, with sequence lengths ranging from approximately 9.8 kb to 28.6 kb. A visual representation of the identified prophages, their length, and location within the host genome is provided in Fig. 3.

#### Prophage genome annotation

**Fig. 4:**
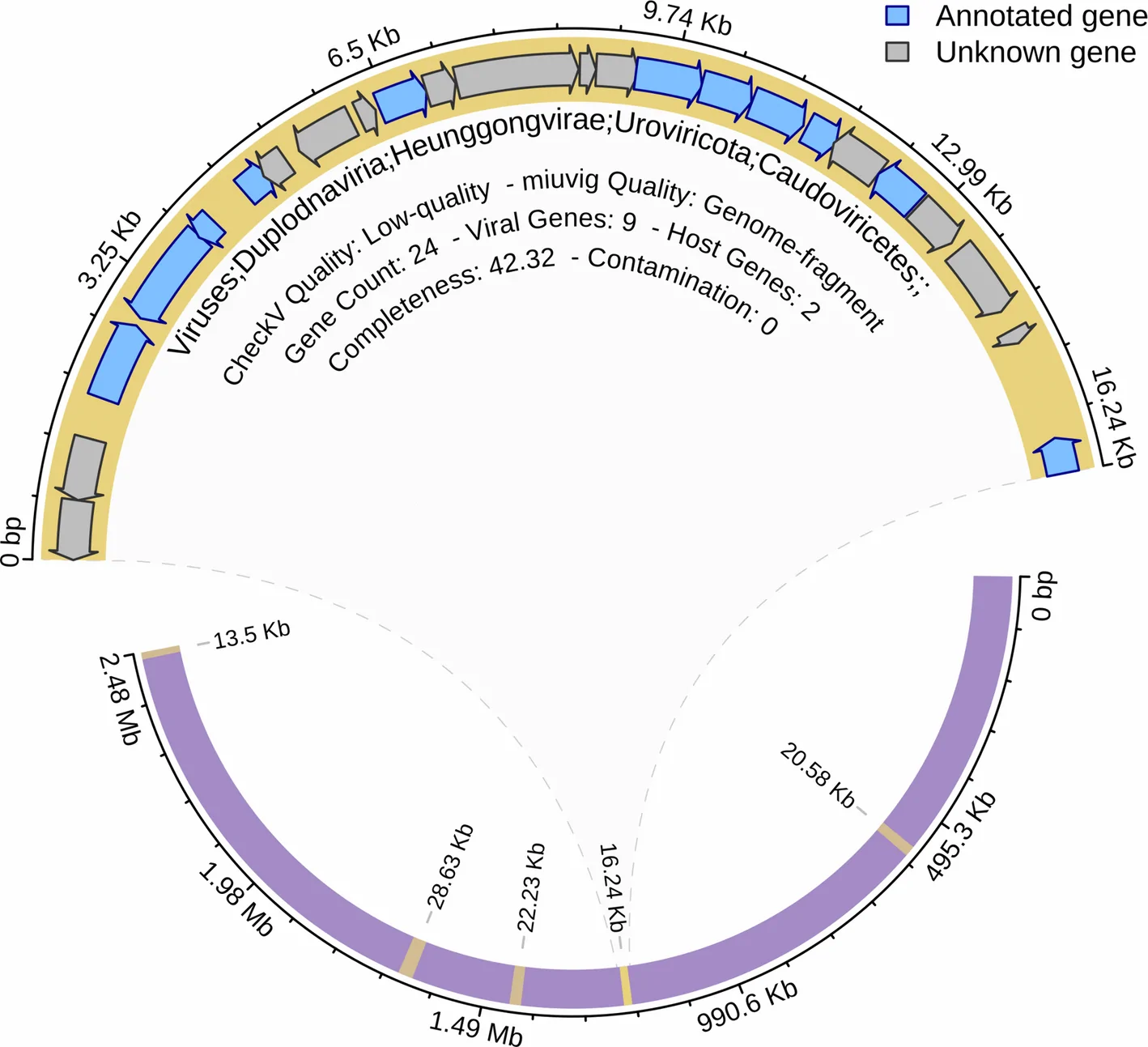
Example prophage feature map generated by the PHI HTML report. For each candidate prophage detected by geNomad, PHI generates a separate report tab summarizing prophage-level information, including sequence coordinates, length, quality classification, taxonomic annotation, predicted genes, and viral hallmark genes. This visualization complements the host-genome ideogram shown in Fig. 3 by providing a gene-level view of individual candidate prophage regions

A visual representation of the prophage genome and features such as quality, number of genes (and viral genes) is shown for each predicted prophage (Fig. 4). The integrated annotation outputs reported 4350 predicted genes (783 marked as viral-hallmark), with the most common annotations being capsid and structural connector proteins. No virulence-associated genes were detected in the candidate prophage sequences using ABRicate with VFDB. VIBRANT-derived AMG annotations were integrated into the PHI report as candidate functional annotations. In the current automated workflow, no additional manual curation or post hoc filtering is performed, thus these predictions should be interpreted cautiously because AMG detection can include false positives, particularly when host-derived metabolic genes occur near predicted prophage regions, when prophage boundaries are uncertain, or when genes have broad metabolic functions that are not specific to viral genomes [24].

#### Impact of MAG quality in prophage recovery

To assess the influence of the MAG quality in the recovery of prophages, we processed an additional three MAGs recovered with varying degrees of quality scores in the MuDoGeR study [21] and their matching reference genomes (Additional File 6 - Table S3). This analysis included MAGs corresponding to *Anaerostipes hadrus*, *Bifidobacterium longum*, and *Bacteroides fragilis.* All MAG and reference sequences were processed with the same PHI workflow, allowing direct comparison of host-genome quality, candidate prophage counts, viral-quality classifications, and sequence-level correspondence between MAG-derived and reference-derived prophage candidates.

As expected, the MAGs showed substantially greater fragmentation than their matched reference genomes. The *B. fragilis* MAG was classified as a high-quality draft with 99.7% completeness, 2.1% contamination, and an N50 of 37.2 kb, compared with 99.9% completeness, 0.7% contamination, and an N50 of 5.2 Mb for the corresponding reference genome. The *B. longum* MAG was also classified as a high-quality draft, with 90.5% completeness, 3.1% contamination, and an N50 of 23.7 kb, compared with 100.0% completeness, 0.3% contamination, and an N50 of 2.4 Mb for the reference genome. The *A. hadrus* MAG showed lower overall quality, with 63.6% completeness, 4.1% contamination, and an N50 of 2.8 kb, whereas the corresponding reference genome had 100.0% completeness, 1.9% contamination, and an N50 of 3.1 Mb.

Candidate prophage counts differed between MAGs and their matched reference genomes. PHI detected six candidate viral/prophage sequences in the *B. fragilis* MAG compared with three in the corresponding reference genome, three candidates in the *B. longum* MAG compared with one in the reference genome, and two candidates in the *A. hadrus* MAG compared with eight in the reference genome. These differences indicate that candidate prophage counts are sensitive to assembly continuity, genome completeness, and the representation of mobile genetic elements in MAGs. In complete reference genomes, candidate prophages were reported as coordinate-defined provirus regions. In contrast, several MAG-derived candidates were reported as independent viral-like contigs with no genomic coordinates, reflecting the fact that fragmented MAG assemblies may contain viral sequences as separate contigs rather than as bounded prophage intervals within longer host contigs.

To determine whether MAG-derived candidates corresponded to prophage regions detected in the matched reference genomes, we performed a sequence-level comparison between geNomad-predicted MAG candidates and geNomad-predicted reference prophage regions. This analysis identified one largely reference-overlapping candidate in the *B. fragilis* MAG, where the MAG-derived sequence showed strong overlap with one of the reference-derived prophage regions. In *(A) hadrus*, one MAG-derived candidate showed partial correspondence to a larger reference prophage region, consistent with incomplete or fragmented recovery of a prophage locus in the lower-quality MAG. In contrast, the *(B) longum* MAG candidates did not show strong sequence-level correspondence to the single candidate prophage detected in the matched reference genome.

The *B. longum* result illustrates an important limitation of interpreting MAG-based prophage analyses using only global genome-quality metrics. Although the *B. longum* MAG was classified as a high-quality draft, prophage recovery was poorer than expected when compared with the matched reference prophage. One MAG-derived candidate prophage, DAITCN010000030.1, was itself classified as high-quality, had a length of 22,421 bp, contained 33 predicted genes and 8 viral genes, but had no reported genomic coordinates. This indicates that high MAG quality and high viral-candidate quality do not necessarily imply recovery of the same prophage region present in the reference genome. MAG quality estimates primarily reflect recovery of conserved genes, whereas prophages are accessory, mobile, and often strain-variable regions. Therefore, a high-quality MAG may still lack the specific prophage locus present in a reference genome, contain a different strain-specific prophage, or assemble viral sequences as separate contigs.

A full accuracy benchmark would require larger numbers of matched MAG/reference pairs, strain-resolved ground truth, and explicit assessment of prophage boundary recovery, sequence overlap, and gene-content conservation. The full results of MAGs versus references is available in the supplementary materials.

## Conclusions

The PHI Toolkit, available through the Galaxy platform, addresses a critical bottleneck in phage research by enabling worldwide access to powerful, yet accessible, computational analyses for both bioinformatics experts and non-experts. Rather than introducing novel predictive methods and algorithms, PHI consolidates tools into a streamlined web interface with a modular, cloud-hosted architecture, eliminating the need for local hardware and advanced bioinformatics expertise. Researchers can therefore avoid manual data wrangling and devote their efforts to biological discovery rather than software installation, maintenance, or resource management. Because each module is version-tracked and can be updated, users can rely on established and actively maintained tools without manual upgrades, while still retaining the flexibility to swap in new tools as the field evolves. Furthermore, the integration of these results within a single interactive report facilitates biological interpretation by linking host identity, prophage content, genome context, and functional annotations. For instance, host genome ideograms and prophage feature maps allow users to examine the genomic location, size, and characteristics of predicted prophages within their bacterial hosts, while iPHoP-derived host-prediction tables provide additional evidence for potential host-phage relationships. When combined with prophage annotations, virulence screening, AMG detection, and host prediction outputs, this integrated structure enables rapid identification of biologically relevant patterns that would be difficult to infer from individual result files alone. Although the interactive HTML report is a central feature of PHI because it links host-level, prophage-level, and interaction-level outputs into a single navigable summary, it is intended primarily as an overview and interpretation layer. For very large analyses involving hundreds of genomes or large numbers of candidate prophages, users may benefit from splitting analyses into biologically meaningful subsets and using exported tabular outputs for downstream filtering, statistical analysis, and custom visualization.

In addition to the complete-genome demonstration, the MAG/reference comparison showed that PHI can also be applied to fragmented metagenome-derived genomes, but also highlighted the effects of MAG fragmentation, genome incompleteness, contig structure, binning behavior, and possible strain-level variation in prophage content. In the current PHI workflow, tRNA-related information is incorporated through the integrated tools rather than through a standalone tRNA-prediction module. geNomad is run with tRNA identification enabled, and iPHoP may use tRNA matches as one of the evidence layers contributing to host-prediction results. PHI therefore reports tRNA-associated evidence when it is produced by these integrated tools, but it does not currently run a tRNAscan-SE module or provide an independent tRNA-focused summary table. A further limitation of the current PHI implementation is that candidate prophage detection relies primarily on geNomad. This provides a reproducible and Galaxy-compatible detection strategy, but it does not eliminate the known uncertainty associated with computational prophage prediction. Different prophage-identification tools use distinct sequence features, reference databases, classification models, and thresholds and may therefore recover partially non-overlapping sets of prophage regions. Highly divergent, short, degraded, or fragmented prophages may remain undetected, particularly in MAG-derived assemblies, whereas plasmids, genomic islands, or other viral-like mobile genetic elements may occasionally generate false-positive predictions. Candidate prophages reported by PHI should therefore be interpreted as computational predictions and evaluated together with viral-quality estimates, viral-gene content, genome context, host-genome quality, and downstream annotation evidence. Future development of PHI will focus on expanding the workflow with additional prophage-detection, host-prediction, annotation, tRNA prediction, attL/attR-site prediction, functional categorization tools such as Prokaryotic Virus Remote Homologous Groups (PHROGs) [25], and visualization modules as new Galaxy-compatible implementations become available. Beyond general phage–host interaction studies, PHI may support applied microbiome research by enabling systematic screening of prophage content, virulence-associated genes, auxiliary metabolic genes, and host defense systems in biotechnologically relevant bacteria. In the future, this could include microbial strains considered for bioinoculants or low-risk pesticide applications [26, 27], provided that dedicated validation datasets are used and the outputs are interpreted as part of a broader strain-characterization and risk-assessment framework.

## Availability and requirements

**Project name**: PHI: Prophage-Host Interaction Toolkit

**Project home page**: https://github.com/Helmholtz-UFZ/galaxy-tools/tree/main/tools/phi-toolkit

**Galaxy workflow**: https://usegalaxy.eu/published/workflow?id=c62d65832377e376

**Operating system(s)**: Platform independent through Galaxy

**Programming language**: Galaxy workflow format; R/R Markdown for report generation; integrated tools use their respective languages

**Other requirements**: No local installation is required when running PHI through Galaxy Europe. Deployment on another Galaxy instance requires installation of the listed Galaxy tools and compatible database versions

**License**: **MIT**

**Any restrictions to use by non-academics**: None

## Supplementary information

Below is the link to the electronic supplementary material.

Supplementary Material 1

Supplementary Material 2

Supplementary Material 3

Supplementary Material 4

Supplementary Material 5

Supplementary Material 6

## Data Availability

The PHI Toolkit consists of the Galaxy workflow itself and a utility Galaxy tool (the finalstep of the workflow) that integrates the results of all used tools. The utility tool is developed at https://github.com/Helmholtz-UFZ/galaxy-tools/tree/main/tools/phi-toolkit and has been deployed to the Galaxy tool shed (which allows installation on anyGalaxy instance). The full set of results of the MBARC22 and MAG datasets, tool, and workflow has been deposited at Zenodo (https://doi.org/10.5281/zenodo.22707434). The MBARC22 genome accessions used for the demonstration are listed in Additional File 2 Figure S2 and were obtained from NCBI, except for NC_010067.1, which was suppressed and therefore excluded.

## Abbreviations

AMG: Auxiliary metabolic gene
ANI: Average nucleotide identity
GTDB: Tk—Genome Taxonomy Database Toolkit
HTML: HyperText Markup Language
MAG: Metagenome—assembled genome
MIMAG: Minimum Information about a Metagenome—Assembled Genome
MISAG: Minimum Information about a Single Amplified Genome
MIUViG: Minimum Information about an Uncultivated Virus Genome
NCBI: National Center for Biotechnology Information
PHI: Prophage—Host Interaction
SAG: Single amplified genome
VFDB: Virulence Factor Database
