# Supplementary material for: PHI: a Galaxy-based workflow for reproducible prophage-host interaction analysis and standardized viral-genomics reporting
Source: BMC Bioinformatics. 2026 Nov 11;27:274. doi: 10.1186/s12859-026-06711-w (PMC13761014; doi:10.1186/s12859-026-06711-w)
Supplement: Supplementary file 2 [file 12859_2026_6711_MOESM6_ESM.docx]

# PHI Toolkit Galaxy Tutorial

**Important: users must log in to their Galaxy account before importing the workflow, uploading files, creating collections, running PHI, or exporting results.**

# 1. Before you start

- Open the Galaxy Europe server in a web browser.
- Log in to your Galaxy account. Running the full workflow, saving histories, importing workflows, and exporting outputs requires an authenticated Galaxy session.
- Prepare the input bacterial genomes or MAGs in FASTA format. Use one FASTA file per genome or MAG.
- For larger projects, consider splitting the dataset into biologically meaningful groups, such as by project, sample type, taxonomic group, treatment, or genome-quality tier. PHI can process many genomes, but a single HTML report may become difficult to navigate for hundreds of genomes.

# 2. Step-by-step instructions

## Step 1 - Import the PHI workflow

Open the published PHI workflow link, then select the option to import or copy the workflow into your Galaxy account. Once imported, the workflow will be available under your saved workflows.

*Figure 1. Importing the PHI workflow into a logged-in Galaxy account.*

## Step 2 - Upload genome or MAG FASTA files

Upload the bacterial genomes or MAGs that you want to analyze. The files should be in FASTA format. After upload, check that every dataset finished successfully and appears in the active Galaxy history.

- Use clear file names, because these names will be propagated into collections and report outputs.
- Large files may take longer to upload and process.

*Figure 2. Uploading bacterial genome or MAG FASTA files to the Galaxy history.*

## Step 3 - Create a dataset list collection

Select the uploaded FASTA datasets and create a list collection. The collection is used as the genome input for the PHI workflow. Make sure the collection contains the intended genomes only.

*Figure 3. Selecting uploaded FASTA datasets and preparing them for a list collection.*

After selecting the files, confirm the list collection name and create the collection. A clear collection name helps when reviewing workflow inputs and outputs later.

## Step 4 - Run the PHI workflow

Open the imported PHI workflow, choose the genome list collection as the input, review all required parameters, and launch the workflow. Galaxy will schedule the workflow steps and show their status in the active history or invocation view.

- Green datasets indicate completed outputs.
- Running or queued jobs may take time depending on server load, input size, number of genomes, and the number of detected prophage regions.
- Memory-intensive steps such as iPHoP may require substantial resources.

*Figure 4. Creating the list collection and selecting it as input when launching the PHI workflow.*

## Step 5 - Open the PHI HTML report in Galaxy

When the workflow finishes, locate the PHI toolkit report output in the Galaxy history or the invocation Outputs tab. Select Preview or open the HTML output to inspect the report in Galaxy.

*Figure 5. Opening the PHI HTML report from the Galaxy workflow outputs.*

## Step 6 - Interpret the report and use tabular outputs for larger analyses

The HTML report is intended as a navigable overview and interpretation layer. It links host genome quality, taxonomy, candidate prophage content, prophage feature maps, host prediction, virulence-factor screening, AMG annotations, and defense-system information. For small to moderate datasets, this provides a convenient way to inspect results in a single document.

For datasets containing hundreds of genomes or very large numbers of candidate prophages, a single HTML report may become large, slower to render, and more difficult to navigate. In these cases, users should split analyses into biologically meaningful subsets and use exported tabular outputs for downstream filtering, statistical analysis, and custom visualization.

## Step 7 - Export the full workflow results

To download or archive the full workflow invocation, open the Export tab in the Galaxy invocation view. Select an export format, choose the destination, generate the download link, and download the exported results when Galaxy reports that the export is ready.

- The Research Object Crate option can be used to preserve workflow information and associated outputs.
- Temporary download links may expire, so download the export promptly.
- For manuscript or supplementary-material preparation, also keep the final HTML report and the relevant tabular output collections.

*Figure 6. Exporting the full Galaxy workflow invocation and downloading the result archive.*

# 3. Troubleshooting

| **Issue** | **Suggested action** |
| --- | --- |
| The workflow cannot be imported | Confirm that you are logged in and that the workflow link is accessible. |
| Input files are not visible when launching the workflow | Check that the files are in the active history and that they were placed into a list collection. |
| Some downstream viral modules are skipped | This can happen when geNomad does not detect candidate viral/prophage sequences for a genome. |
| The HTML report is slow or difficult to navigate | Use smaller grouped runs and analyze exported tabular outputs outside the report. |
| The export link expired | Return to the Export tab and generate a new download link. |
